# National and international models of involving people with lived experience in dementia policy, advocacy and research

**DOI:** 10.3389/frdem.2026.1816031

**Published:** 2026-06-02

**Authors:** Ellen Snowball, Robin Casey, Karen Myers Barnett, Sarah Campill, Jan Runar Eliassen, Marion Tur Eliassen, Brian Anderton, Claire Stockwell-Lance, Mary Warbelow, Juanita-Dawne Bacsu, Emma Lanza, Jennifer Bethell

**Affiliations:** 1 KITE Research Institute, Toronto Rehabilitation Institute, University Health Network, Toronto, ON, Canada; 2 Engagement of People with Lived Experience of Dementia Program and Advisory Group, Canadian Consortium on Neurodegeneration in Aging, Toronto, ON, Canada; 3 Alzheimer Europe, Senningerberg, Luxembourg; 4 Alzheimer’s Society, London, United Kingdom; 5 Global Brain Health Institute, Trinity College Dublin, Dublin, Ireland; 6 School of Nursing, Thompson Rivers University, Kamloops, BC, Canada; 7 Lady Davis Institute, CIUSSS du Centre-Ouest-de-l’Île-de-Montréal, Montréal, QC, Canada; 8 Institute of Health Policy, Management & Evaluation, Dalla Lana School of Public Health, University of Toronto, Toronto, ON, Canada

**Keywords:** advisory group, advocacy, dementia, health research, lived experience, partnership-oriented, patient and public involvement, patient engagement

## Abstract

Globally, there is growing recognition of the need to advance approaches to involve people with lived experience of dementia as collaborators in policy, advocacy and research activities. Involvement is viewed as a right by dementia advocates and others, and some organizations have developed mechanisms to support this collaboration, such as through dedicated resources for infrastructure or as a condition of research funding. However, there is limited literature on how national and international organizations support the involvement of people with lived experience of dementia. In this perspective article, we describe different approaches to involving people with lived experience in policy, advocacy and research activities across national and international network contexts. We outline and compare the approaches taken by the Engagement of People with Lived Experience of Dementia program and advisory group (Canadian Consortium on Neurodegeneration in Aging), Alzheimer Society Research Network (Alzheimer’s Society UK), European Working Group of People With Dementia and European Dementia Carers Working Group (Alzheimer Europe), and the Lived Experience Advisory Group (Global Brain Health Institute, Trinity College Dublin). For each example presented, we describe the initiative (e.g., purpose, brief history, structure). We discuss the four initiatives in order to identify common and context-specific barriers and enablers to involvement. We hope that the findings will help others to develop their own initiatives to involve people with lived experience of dementia.

## 1 Introduction

Patient involvement, engagement and partnerships are terms used internationally to describe research and related activities ‘with’ or ‘by’ individuals with personal experience of a health issue and their family, friends and caregivers, rather than ‘to’, ‘about’ or ‘for’ them (Alzheimer’s Society, n.d.; Carman et al., 2013; Sheridan et al., 2017; Gove et al., 2018; Greenhalgh et al., 2019). However, there are sometimes ambiguities or inconsistencies in how these terms are used. In particular, the term “patient” has been critiqued for failing to account for people’s full identities and experiences and underemphasizing inclusion, partnership, and shared ownership (SPOR, n.d.; Alzheimer Europe, 2019; Georges et al., 2022). The terms used to describe these collaborative approaches also vary across countries and disciplines. ‘Engagement’ and ‘involvement’, for example, have developed independently in different countries and contexts (Georges et al., 2022). Consequently, in one framework, ‘involvement’ is the overarching concept (UK Standards for Public Involvement, n.d.) and, in another, ‘involvement’ represents a sub-type of ‘engagement’ (Manafò et al., 2018). Similarly, in the context of research, these concepts also overlap with other approaches, for example, integrated knowledge translation (Nguyen et al., 2020a) or participatory research (Jull et al., 2017) but can differ in regard to motivations, approaches and principles. In this article, we will refer to involving/involvement of people with lived experience to describe these activities collectively and without reference to an underlying framework or orientation, unless otherwise specified. As an international authorship, respecting each group’s practices and preferences, each contributing group will also use their own locally-accepted terminology when describing their model of involvement.

Involving people with lived experience has distinct implications for policy, advocacy and research related to dementia. Dementia is stigmatized (Link and Phelan, 2001; Herrmann et al., 2018; Bacsu et al., 2022, 2025) and for people with lived experience of dementia, this can create barriers (e.g., public-stigma and self-stigma creating assumptions about inability to contribute) (Nguyen et al., 2020b) as well as motivations for involvement [e.g., challenging stigmas, making meaning from experience (Snowball et al., 2022)]. Recommendations for tailored approaches to involving people with lived experience of dementia have been described (Alzheimer Europe, 2019, 2022; Snowball et al., 2024; Walter et al., 2025) and reviews have summarized activities in the contexts of policy, advocacy and research, (Bethell et al., 2018a; Burton et al., 2019; Innes et al., 2021; Groothuijse et al., 2024; Bühler et al., 2025). However, less is known about the organizational supports that develop and sustain involvement.

The objective of our paper is to describe and compare involvement initiatives across national and international contexts: Engagement of People with Lived Experience of Dementia program and advisory group (Canadian Consortium on Neurodegeneration in Aging), Alzheimer Society Research Network (Alzheimer’s Society UK), European Working Group of People With Dementia and European Dementia Carers Working Group (Alzheimer Europe), and the Lived Experience Advisory Group (Global Brain Health Institute, Trinity College Dublin). These international groups have articulated a common interest in involving people with lived experience of dementia in dementia policy, advocacy and research. Three of our four organizations collaborated on an Immersive workshop about involvement at the July 2025 Alzheimer’s Association International Conference (AAIC), which resulted in the conception and drafting of this article. This article was co-authored by researchers, people with lived experience and program managers who have participated in planning, conducting, and evaluating various involvement initiatives. Our aim is not to describe individual experiences of involvement [although we offer examples of such narratives through our and others’ work (Alzheimer’s Society, 2019; Global Brain Health Institute, 2022; Alzheimer Europe, 2025)]. Our aim is to outline and compare the approaches of our different organizations, while discussing barriers and enablers to involving people with lived experience. Our hope is to build knowledge of different approaches to involvement in order to stimulate advancements in this area. The findings from this article will have implications for researchers, knowledge users (e.g., policy makers, research funding organizations and health charities), and people with lived experience working to develop collaborative initiatives in dementia policy, advocacy and research.

## 2 Four models of involvement

Descriptions of the four models of involvement are summarized in Table 1 and discussed below.

**Table 1:** Description of engagement/involvement initiatives from four national and international networks.

Name & website	Country	Year established	Focus	Organization	Funding	Involvement approach	Recruitment strategies	Support
Engagement of People with Lived Experience of Dementia (EPLED) www.epled.ca	Canada	2019	Research	Canadian Consortium on Neurodegeneration in Aging (CCNA)	Canadian Institutes of Health Research, Alzheimer Society of Canada	EPLED Advisory Group (~15 people with dementia, friends, family caregivers/care partners)	Online (e.g., website) Strategy for Patient Oriented Research (SPOR) support units Informal networks Alzheimer societies CCNA network	2 academic co-leads (researchers) 2 staff (1 full time and 1 part time) Monthly meetings Training Honoraria paid annually and through project engagements Travel expenses paid (including for support person, as required) for EPLED activities (e.g., CCNA annual meeting)
Alzheimer Society Research Network (RN) www.alzheimers.org.uk	United Kingdom	1999	Research	Alzheimer’s Society UK	Voluntary charitable income	The Research Network (~185 people with dementia, carers and former carers)	Online - Website and careers job board Internal bulletins, e.g., Involvement Bulletin External events Volunteer promotion events Informal networks	Senior Involvement Partner (part-time) Involvement Lead (Part-time) Research Involvement Officer (full-time) Allocated role manager to support volunteering journey Bi-monthly meetings (usually 1 in-person event) Induction and Training Expenses paid (travel, accommodation & subsistence) Regular communications and updates Access to external wellbeing support and advice Organizations volunteering team (including support through volunteer policies)
European Working Group of People With Dementia (EWGPWD) www.alzheimer-europe.org	Europe	2012	Research, policy & advocacy	Alzheimer Europe	To coordinate the activities and meetings of the EWGPWD, AE receives public grants at EU and national level (e.g., Citizens, Equality, Rights and Values and Horizon Europe programs, Luxembourg National Research Fund). The work of the working group is also supported with Public Involvement activities with private companies.	EWGPWD (~15 people with dementia)	People with dementia can be nominated by their national Alzheimer associations (who are member organizations of AE).	All Public Involvement and Ethics team members (5 full-time) coordinate and support EWGPWD members (online and in-person). Regular meetings (usually 2 in-person and 3 online meetings per year). All expenses of members and their supporters (a person of their choice) linked to attending in-person meetings or events are covered by AE. Members of the EWGPWD do not receive honoraria from AE.
European Dementia Carers Working Group (EDCWG) www.alzheimer-europe.org	Europe	2022	Research, policy & advocacy	Alzheimer Europe	To coordinate the activities and meetings of the EDCWG, AE receives public grants at EU and national level (e.g., Citizens, Equality, Rights and Values and Horizon Europe programs, Luxembourg National Research Fund). The work of the working group is also supported with Public Involvement activities with private companies.	EDCWG (~15 current and former carers)	Current carers, relatives and supporters of people with dementia or carers with prior experience of caring in the 5 years prior to their nomination can be nominated by their national Alzheimer associations (who are member organizations of Alzheimer Europe).	All Public Involvement and Ethics team members (5 full-time) coordinate and support the EDCWG members (online and in-person). Regular meetings (usually 2 in-person and 3 online meetings per year.). All expenses of members linked to attending in-person meetings or events are covered by AE. Members of the EDCWG do not receive honoraria from AE.
Lived Experience Advisory Group www.gbhi.org	Ireland	2020	Research, Advocacy	Global Brain Health Institute – Trinity College Dublin	Trinity College Dublin	Lived Experience Advisory Group (~4–5 people with dementia, friends, family caregivers/care partners)	Online (GBHI website) Member recruitment Leaflets (circulated at international conferences) Local and international events	GBHI Site Director Trinity 1 GBHI Staff (full-time) Bi-monthly meeting All expenses of members linked to attending in-person meetings or events are covered by GBHI Opportunities for engagement with GBHI fellows Opportunities for engagement GBHI curriculum and events

### 2.1 Engagement of People with Lived Experience of Dementia (EPLED) program and advisory group—Canadian Consortium on Neurodegeneration in Aging (CCNA)

The Canadian Consortium on Neurodegeneration in Aging (CCNA) was initiated in 2014 to advance research on neurodegenerative diseases (Chertkow et al., 2024). It is a pan-Canadian network made up of clinicians, researchers, trainees and other partners working in the areas of prevention, treatment, and quality of life and across all types of research (i.e., biomedical, clinical, health services and population health). In CCNA Phase I (2014–2019), there was no program dedicated to engaging people with lived experience, however, there were projects involving people with lived experience [e.g., as patient engagement (Bethell et al., 2018b) and community-based research (Jacklin et al., 2020)]. The Engagement of People with Lived Experience of Dementia (EPLED) program was established in CCNA Phase II (2019–2024) through dedicated funding from the Alzheimer Society of Canada. EPLED’s objectives are to: (1) Support persons with dementia and care partners to be involved in the research process; (2) Work with research teams and partners to develop novel mechanisms to further this collaboration; and (3) Advance the methods of patient engagement in research through evaluation. Now, in CCNA Phase III (2024–2029), EPLED functions as a core component of CCNA through its Dementia Research Support Hub.

EPLED’s main activity is to support an Advisory Group, composed of people with lived experience (i.e., people living with dementia, family and current or former caregivers/care partners), recruited from across Canada, to be engaged in research. This model was developed with the intention to support CCNA researchers working across all themes of research (Bechard et al., 2022). EPLED Advisory Group members are recruited to balance perspectives and experiences including role (e.g., care partner/care giver), type of dementia, gender, location (within Canada) and, when applying to the Advisory Group, applicants are asked about other perspectives they may have (e.g., backgrounds, identities, abilities, etc.). Advisory Group members are involved in research activities both internal and external to CCNA, such as: (1) Providing feedback at research meetings and other activities (Boucher, 2022); (2) Developing academic (e.g., Bethell et al., 2021; Rojas-Rozo et al., 2024; Smith et al., 2025) and non-academic (Gauthier et al., 2022) resources for diverse audiences (3) Presenting at academic and non-academic meetings and events (Snowball et al., 2024); (4) Contributing to CCNA research teams as co-investigators (Lemay-Compagnat et al., 2025) and; (5) Co-developing an evaluation framework to assess EPLED’s implementation and impacts. EPLED contributions to CCNA events (e.g., annual conference and public events) have been brought about through strong partnership with the CCNA Knowledge Mobilization program; EPLED members deliver opening and closing remarks, act as panel discussants, host art exhibitions (Government of Canada, CIHR, 2025; Snowball et al., 2026) and, most recently, EPLED developed and adjudicated a poster prize, named for Wayne Hykaway (an inaugural Advisory Group member), to encourage and recognize trainee skills in communicating with non-academic audiences. Throughout, engagement in specific research and program activities is flexible and at the discretion of the individual Advisory Group member, based on their own interests and recognizing their changing circumstances.

### 2.2 Alzheimer Society Research Network (RN)—Alzheimer’s Society UK

Alzheimer’s Society UK is a dementia charity committed to improving the lives of people affected by dementia. Through support, research, and campaigning for better experiences, Alzheimer’s Society UK have funded dementia research and prioritized involving people with lived experience in decision-making. In 1999, they established the Research Network (RN), an Advisory Group of 185 volunteers with lived experience of dementia (i.e., people with a diagnosis of dementia, current/former carers) (Corner, 2002; Alzheimer’s Society, 2014, n.d.). These volunteers shape research to ensure it is relevant, credible and impactful. They contribute at every stage of the research process, from reviewing applications to supporting dissemination. Their involvement strengthens research quality and promotes awareness and understanding of dementia, helping to drive meaningful change for those affected.

The RN plays a vital role in shaping the Alzheimer’s Society UK research program. Alongside expert scientific and medical panels and peer reviewers, members contribute at every stage of the grant funding process, including shortlisting applications, supporting project development, reviewing and scoring proposals, interviewing fellowship candidates, and attending funding panels. Beyond funding decisions, RN members collaborate with researchers throughout research projects, offering insights that enrich study design, public engagement, and dissemination plans. They participate in advisory groups, co-create resources, and help ensure research remains relevant and inclusive. Alzheimer’s Society UK prioritizes early career researchers, and the RN has developed workshops, guidance documents, and practical tools to support them. For example, they created resources explaining how to involve people with lived experience effectively and how to foster meaningful connections (UCLH Biomedical Research Centre, Alzheimer’s Society and Parkinson’s UK, n.d.). They have also extended their influence to funding programs for grant funders including the Medical Research Council, Engineering and Physical Science Research Council, and Marie Curie. Their involvement ensures that dementia research reflects the perspectives of those most affected, driving innovation and impact across the research landscape.

### 2.3 European Working Group of People With Dementia (EWGPWD) and European Dementia Carers Working Group (EDCWG)—Alzheimer Europe

Alzheimer Europe (AE) is an umbrella organization that includes 41 national Alzheimer Associations from 36 European countries. AE’s overarching aim is to change perceptions, policy and practice to improve the lives of people affected by dementia. AE has long prioritized the active involvement of people with dementia in its activities and, in particular, in dementia research. Such involvement, which stretches back to 2003, initially took place on a more informal and occasional basis, and has been progressively strengthened and broadened over time (Roberts et al., 2020; Georges et al., 2022). A major step in this process was the establishment of the European Working Group of People With Dementia (EWGPWD) (Alzheimer Europe, n.d.a) in 2012, followed a decade later, in 2022, by the creation of the European Dementia Carers Working Group (EDCWG) (Alzheimer Europe, n.d.b). The working groups involve people with lived experience (i.e., 15 people with dementia and 15 carers respectively) who are nominated by their national Alzheimer Association and serve three-year terms. Members of the working groups contribute to the work and activities of the organization and meet regularly online and in person. The Chairperson of each working group also serves as an ex-officio member of the AE Board with full voting rights. Within these structures, members primarily contribute to research (Diaz et al., 2021) and policy activities through Public Involvement (PI) (Alzheimer Europe, n.d.c) and reflections related to ethics. Additionally, they contribute to advocacy activities and campaigns, such as the European Parliament election campaign (Alzheimer Europe, 2023a, 2024) to which members contributed by engaging with policymakers and sharing their priorities and experiences to inform European political commitments.

AE’s PI model is grounded in people’s right to voice their needs and perspectives and in democratic processes such as legitimation and transparency. Researchers, in turn, benefit from the lived experience and unique perspectives of contributors, which can strengthen research processes, improve outcomes and reframe priorities. AE’s model is flexible and adapts and responds to the needs, possibilities and interests of a diverse group of people with lived experience. While there is no single way to do PI, there are key principles that ensure it is ethically conducted (Gove et al., 2018; Alzheimer Europe, 2019, 2022). For instance, PI must be meaningful and timely, ensuring that people receive accessible information, that their contributions are recognized and their autonomy respected, and that they can participate safely and confidently in ways that genuinely influence the research process. Equally important is a commitment to diversity, which requires listening to and learning from people with different experiences, and the reduction of structural barriers (e.g., rigid recruitment methods, inaccessible communication formats, inflexible study requirements) (Alzheimer Europe, 2021, 2023b). This is reflected in the quality of relationships, which need to be built and nurtured so that shared learning and mutual respect can develop. The art lies in the continuous effort to identify and mitigate imbalances, allowing members to be respected and supported according to their needs.

### 2.4 The Lived Experience Advisory Group—Global Brain Health Institute (GBHI), Trinity College Dublin

Global Brain Health Institute (GBHI) is an organization aiming to improve brain health across the world by: (1) Providing training to international fellows through the Atlantic Fellows for Equity in Brain Health program; (2) Collaborating with transdisciplinary teams on brain health prevention and interventions and; (3) Advocating and sharing knowledge across communities. The Lived Experience Advisory Group at GBHI is an international panel of individuals directly affected by dementia who share their insights to strengthen dementia-related research and practice. Formed within GBHI’s Person and Public Voice (PPV) program, the group plays a central role in shaping dementia-related research, policy, and advocacy by ensuring that people with lived experience inform every stage of GBHI’s work. Its members include people living with dementia, care partners, family members, and others. The group collaborates closely with GBHI faculty, staff, and Atlantic Fellows for Equity in Brain Health to support Lived Experience training, capacity building, and co-creation of research and educational materials, such as the GBHI Dementia Community – Research Advisory Panel (DC-RAP) (n.d.).

Guided by GBHI values—authenticity, fairness, openness, respect, courage, and empathy—the core objectives of the group are to: (1) Shape research priorities: the group reviews and evaluates research proposals and formally provides input on “setting priorities” within GBHI and its PPV program, ensuring that dementia research reflects real-world needs rather than purely academic agendas; (2) Guide research studies: the group contributes directly to research proposals, curriculum, and management, working alongside faculty to review projects and improve how studies are conducted. This includes advising on study design and implementation, including feasibility, participant experience, ethical considerations in dementia research as well as sharing lived experience in clinical and classroom settings; (3) Inform dissemination efforts: the group contributes to the development of training materials, educational resources, and communications to ensure clarity, inclusivity, and relevance. For example, the group co-creates curriculum sessions and reviews fellows’ projects, including guidance on language and imagery to ensure outputs are accessible and non-stigmatizing (e.g., GBHI’s dementia-friendly language guide) (Global Brain Health Institute, 2026a); and (4) Make real-world impact: the group participates in advocacy by highlighting priorities identified through lived experience and by promoting equitable, person-centered approaches in dementia policy discussions. Involvement has directly catalyzed public-facing initiatives, for example, a single discussion with the panel member during a GBHI Ask the Experts session inspired the development of a major Human Rights Day event on dementia (Global Brain Health Institute, 2026b). Over time, the group has become a trusted partner in planning, conducting, and assessing research through sustained collaboration, capacity building, and shared commitment to GBHI’s values. These efforts have positioned the Lived Experience Group as a key driver of meaningful, community-informed change in dementia research and practice.

## 3 Collective learnings

Our groups’ purposes in involving people with lived experience span research, advocacy and policy. Collectively, underpinning all our initiatives, is the imperative to center the voices of people with lived experience of dementia in the activities that impact them, thereby challenging the stigmas associated with dementia and improving the health and quality of life of people living with dementia and their families. Each of our groups is supporting involvement by building and sustaining one or more groups of people with lived experience and facilitating opportunities to collaborate with partners (e.g., researchers, policy-makers and/or trainees). The size of these groups range from 5 to nearly 200, with different approaches (e.g., committee with regular meetings or networks for information sharing) and compositions (i.e., shared or separate groups for people living with dementia and care partners).

Common across all of our groups were challenges related to meeting and group structure impacting membership diversity (e.g., online meetings that require computer literacy and reliable internet connection, language skills that enable participation); lack of resources for program evaluation and knowledge mobilization (e.g., describing implementation and impacts of involvement); and challenges for involvement in some types of research (e.g., in biomedical or preclinical research). Some enablers to involvement were context-dependent, each of the groups addressed these challenges by ensuring the sustainability of key inputs including financial resources (i.e., ongoing funding for program activities), dedicated staff and broad organizational and sector support (i.e., larger network leadership, governmental) for involvement. Other important enablers were the development of supportive and accessible environments, including accessible communication formats, flexible meeting structures, and sufficient preparation time. These were critical in ensuring that people with lived experience were able to participate effectively and meaningfully. Equally important was the adoption of partnership-based approaches that recognized individuals with lived experience as collaborators rather than symbolic representatives. Such approaches helped to foster trust and strengthen collaborative relationships between people with lived experience and professionals.

Still, while this article is a representation of our four networks, we also recognize that our work is not representative of other national and international contexts or involvement initiatives. Involvement in research on dementia has been most commonly reported in studies from the UK, Canada and the United States (Bethell et al., 2018a), where there are structures to support involvement [e.g., INVOLVE (UK), Strategy for Patient-Oriented Research (Canada) and Patient-Centered Outcomes Research Institute (US)], and less is known about approaches to involvement in other international settings. In particular, the broader literature on involvement in research within low and middle income countries highlights diverse applications but global differences in terminology, resources, requirements and reporting (Cook et al., 2019) For involvement in dementia policy, advocacy and research, the role of GBHI is particularly important in developing capacity worldwide through their Atlantic Fellows for Equity in Brain Health Program (see section 2.4.).

## 4 Conclusion

In this perspective article, we described different approaches to involving people with lived experience of dementia in policy, advocacy and research activities across national and international network contexts. Each group’s approach reflected unique roles and resources, and our experiences revealed common challenges, opportunities, and strategies for meaningful involvement. While these models operate within distinct national and institutional contexts, they demonstrate common goals for involvement as well as the importance of establishing structures to enable meaningful and sustained involvement.

Several interrelated implications for best practice emerge from these examples. First, meaningful involvement requires early and continuous participation throughout the policy, advocacy or research processes, rather than consultation at later stages. Second, organizations must invest in supportive infrastructures, resources, and training mechanisms that enable both people with lived experience and professionals to participate effectively in collaborative processes. Third, among people with lived experience, involvement activities should have benefits at the individual (e.g., learning) and group (e.g., solidarity, mutual support) levels to ensure broader impact. Fourth, recognizing lived experience as a form of expertise can enhance the relevance, quality, and societal impact of research, policy and advocacy. Fifth, involvement initiatives should develop and sustain robust evaluation plans to assess impacts for individuals (including but not limited to people with lived experience) as well as in shaping policy, advocacy and research decisions; this will help to build the evidence for effective approaches to involvement, including strategies for addressing shared challenges. Finally, the importance of diverse perspectives and lived experiences, related to dementia as well as demographic and related factors (e.g., social determinants of health), must be acknowledged through context-specific recruitment strategies, however, we also recognize the need for resources to foster this diversity as a matter of equity and social justice (Shimmin et al., 2017).

Involving people with lived experience of dementia is essential to ensuring that dementia policy, advocacy and research prioritize the needs of those most impacted. Embedding meaningful involvement has the potential to strengthen both the legitimacy and effectiveness of these initiatives. Continued learning from international models of practice will be essential for developing inclusive, participatory approaches that place the voices and expertise of people with lived experience at the center of dementia research, policy and advocacy. Moreover, collaboration between international partners is critical to leveraging expertise and building capacity to advance involvement across contexts. By working in global collaboration, we can share knowledge and expertise to help others implement and create best practices to advance meaningful involvement of people with lived experience of dementia.

## Data Availability

The original contributions presented in the study are included in the article/supplementary material, further inquiries can be directed to the corresponding author.
